# Stress granules and cell death: crosstalk and potential therapeutic strategies in infectious diseases

**DOI:** 10.1038/s41419-025-07800-z

**Published:** 2025-07-05

**Authors:** Huan-Shao Huang, Lan Chen, Jia-Xin Chi, Shi-Ying Lai, Jiang Pi, Yi-Ming Shao, Jun-Fa Xu

**Affiliations:** 1 Dongguan Key Laboratory of Pathogenesis and Experimental Diagnosis of Infectious Diseases, The First Dongguan Affiliated Hospital, Guangdong Medical University, Dongguan, China; 2 Institute of Laboratory Medicine, School of Medical Technology, Guangdong Medical University, Dongguan, China; 3 Songshan Lake Innovation Center of Medicine & Engineering, Guangdong Medical University, Dongguan, China

**Keywords:** Infectious diseases, Infectious diseases

## Abstract

Pathogens exploit cellular stress responses to drive infection and evade immune responses, posing a persistent global health threat. Stress granules (SGs), dynamic mRNA hubs formed under stress, and regulated cell death (RCD) pathways collectively orchestrate host-pathogen dynamics. While SGs regulate mRNA translation to aid adaptation, RCD mechanisms—including apoptosis, pyroptosis, and necroptosis—eliminate infected cells to curb pathogen spread. However, pathogens subvert these systems through immune evasion strategies, such as disrupting SGs assembly or hijacking cell death signaling, to enhance replication and persistence. This review integrates molecular insights into SGs biogenesis and RCD regulation, dissecting their bidirectional interplay during infection. We highlight pathogen-specific tactics, such as viral proteases cleaving G3BP1 or bacterial effectors halting translation, to manipulate SGs dynamics and cell death pathways. Furthermore, we explore therapeutic opportunities targeting SGs assembly (e.g., eIF2α phosphorylation modulators, G3BP1 inhibitors) and RCD modulation (e.g., PANoptosis suppression, ferroptosis inducers) to restore host defense and mitigate immunopathology. By bridging molecular mechanisms with clinical applications, this analysis charts a course toward precision therapies leveraging the SGs-RCD axis to combat infectious diseases.

## Facts

- SGs protect host cells by sequestering viral components but may trigger regulated cell death (RCD) under chronic stress, influencing infection outcomes and tissue damage.
- A bidirectional interaction exists between SGs and cell death, where SGs inhibit apoptosis/pyroptosis by sequestering key factors, while DAMPs released during cell death promote SGs assembly, impacting host-pathogen dynamics.
- Combined targeting of SGs and cell death pathways (e.g., via eIF2α or G3BP1 modulation) offers dual benefits, enhancing antiviral efficacy while reducing inflammation, with potential for precision medicine in infectious diseases.
- Emerging cell death forms (ferroptosis, cuproptosis, PANoptosis) exhibit unique roles in infection and may serve as future therapeutic targets for pathogen clearance and tissue protection.
- Pathogens manipulate SGs assembly and function (e.g., degrading G3BP1 or hijacking translation factors) to evade immunity and enhance replication, highlighting the need for mechanistic studies to counteract these strategies.

## Open questions

- How do specific pathogens differentially manipulate SGs assembly and disassembly to enhance their survival and replication?
- What are the molecular mechanisms underlying the bidirectional interaction between SGs and RCD pathways during infection?
- Can the dynamic balance between SGs and cell death be therapeutically targeted to restore host defense without causing excessive tissue damage?

## Introduction

Infectious diseases are a major global public health issue. Since COVID-19 outbreak, nearly 770 million cases and 7 million deaths have been reported, with virus mutations and recurrences complicating control. Traditional diseases like tuberculosis and malaria face drug resistance, while emerging diseases like MERS, Zika, and monkeypox have high fatality rates [1]. Traditional treatments have limitations like long detection times, low sensitivity, and difficulty detecting rare pathogens [2]. Antibiotics are less effective against viruses and drug-resistant bacteria [3]. Therefore, developing new treatment strategies like precision medicine, novel antibiotics, immunotherapy, nanotechnology and so on, is crucial to tackle drug resistance and emerging pathogens.

At the cellular level, infectious diseases can trigger multiple stress responses, including the formation of stress granules (SGs). When eukaryotic cells face adverse conditions such as hypoxia, heat stress, oxidative stress, osmotic stress, and viral infection, stress responses are activated to mitigate damage [4]. During this process, a reversible dynamic structure known as SGs forms in the cytoplasm. SGs are aggregates of ribonucleoprotein (RNP) complexes, primarily composed of untranslated mRNAs, ribosomal subunits, translation initiation factors, and RNA-binding proteins [5]. SGs help cells survive and maintain function under by regulating mRNAs translation, degradation, or storage. However, abnormal formation and breakdown of SGs are associated with pathologic conditions, including cancer, increased viral infections, and neurodegenerative diseases [6].

Cell death, including Necrosis and programmed cell death (also known as regulated cell death, RCD), is essential for maintaining homeostasis and organism development. RCD includes apoptosis, pyroptosis, necroptosis, ferroptosis, and cuproptosis, all regulated by signaling pathways and molecular mechanisms under various conditions [7]. In infectious diseases, these cell death forms help control infections by clearing infected cells and limiting pathogen replication [8]. However, excessive or uncontrolled cell death can cause tissue damage and inflammatory, worsening the disease.

Currently, the mechanisms by which SGs act in cell death modes remain incompletely understood. SGs formation often seen as a protective mechanism, can be triggered by pathogen invasion or the host immune response during infection. By sequestering viral RNA and proteins, SGs protect host cells, inhibit viral replication, reduce inflammatory, and prevent cell death [9]. However, persistent SGs may sometimes disrupt cellular homeostasis and induce cell death [10].

The interaction between SGs and cell death in infectious diseases is complex. Therefore, this review aims to provide a comprehensive overview of the structure and assembly mechanisms of SGs, the types and regulation of cell death, and further explore the specific mechanisms of SGs and cell death, offering new perspectives for developing novel anti-infection strategies.

## Formation and regulation mechanism of SGs

### Formation mechanism of SGs

SGs formation is dynamically regulated by cellular context, stressor-specific triggers, and signaling networks, creating an adaptable response to diverse stressors [11]. Key components of SGs include untranslated mRNAs, translation initiation factors (e.g., eIF4E, eIF3, p-eIF2α), RNA-binding proteins (e.g., TIA-1), proteins related to mRNA metabolism (e.g., G3BP, FAST), signaling proteins (e.g., mTOR), and regulatory proteins [4]. These components form the core network of SGs through intricate protein-protein, protein-RNA, and RNA-RNA interactions, collectively regulating their assembly and function, with the G3BP1 complex as a key driver [12]. G3BP1 drives SGs assembly via liquid-liquid phase separation, with key structural features including the N-terminal nuclear transport factor 2 (NTF2) domain, the C-terminal RNA-binding domain (RBD), and intrinsically disordered region (IDR) [13]. Research shows that the absence of G3BP1/2 or other important components did not completely inhibit SGs formation [4]. G3BP1 mutants lacking the NTF2 or RBD domains cannot mediate SGs assembly, while IDR1 and IDR2 are not essential for function [14]. Additionally, Caprin-1 and USP10 promote or inhibit SGs formation, respectively, by interacting with the NTF2L domain of G3BP1 [15].

**Fig. 1:**
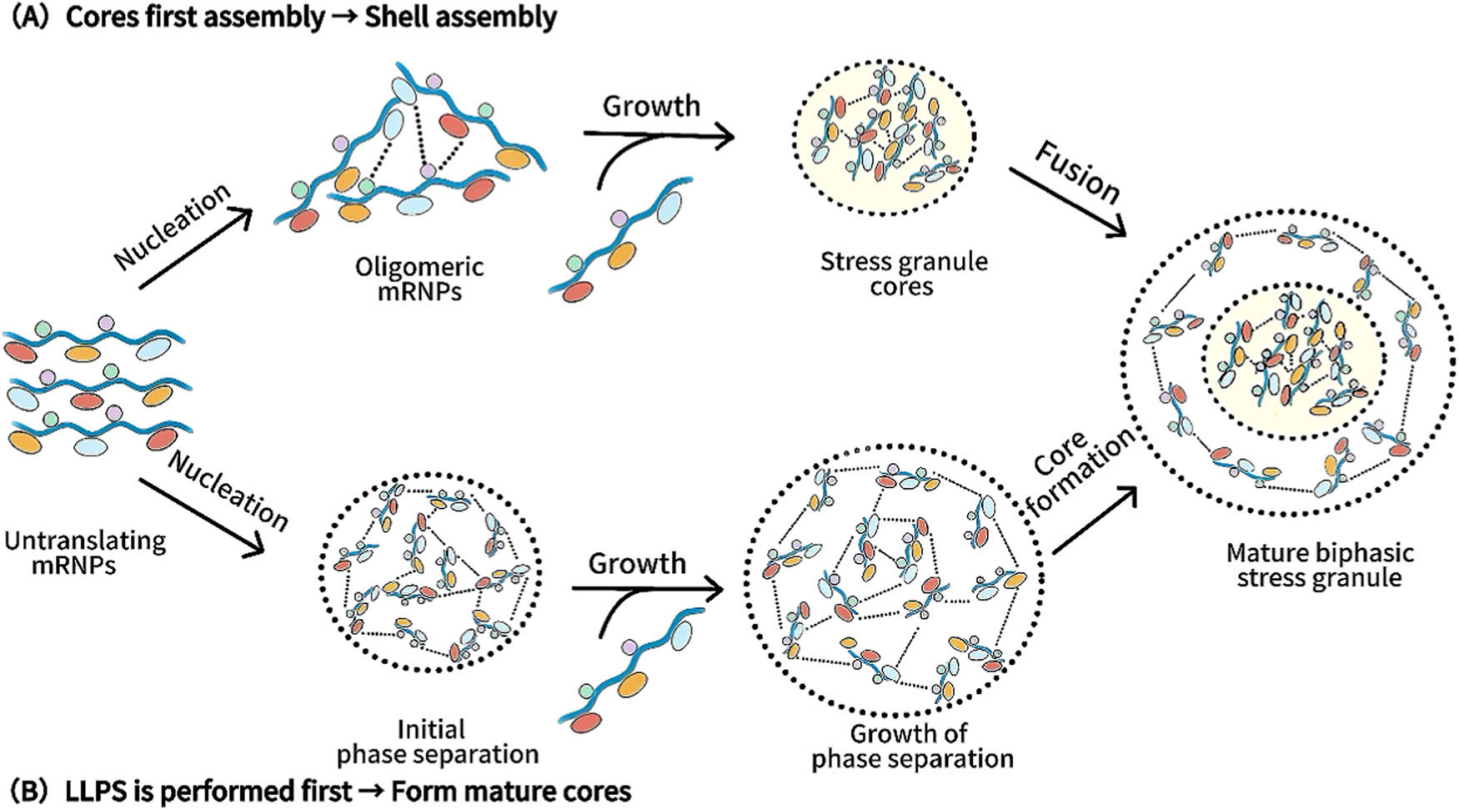
Two models of discrete phases in SGs assembly (Created with huashijie.art).

Super-resolution microscopy and fluorescence recovery after photobleaching experiments show that SGs have a dichotomous structure with a stable “core” and a dynamic “shell”, allowing continuous exchange of components with surrounding cellular compartments, with rapid exchange in the shell and slower exchange in the core [16]. Two models describe SGs assembly, as shown in Fig. 1. In the “core-first” model, translation-suppressed RNPs aggregate into oligomers as nucleation sites, then more RNPs are added to form larger dynamic shells. In the “liquid–liquid phase separation” model, SGs form via weak protein-nucleic acid interactions. Translation-inhibited RNPs form droplets, grow by combining with concentrated RNPs, and stabilize into mature SGs when protein concentration exceeds the threshold [17]. Recent researches highlight the role of membrane structures in SGs assembly. The endoplasmic reticulum (ER) provides a platform for SGs assembly, promotes SGs division via its lumen, and exchanges mRNA with SGs to regulate translation and stress adaptation. SGs also enhance antiviral defenses by interacting with MAVS on mitochondrial membranes and influencing mitochondrial unfolded protein responses [9]. These findings advance our understanding of SGs assembly mechanisms and open new avenues for research into SGs regulation and therapeutic strategies.

### Dynamic regulation of SGs

The integrated stress response (ISR) activates eIF2α phosphorylation, suppressing translation initiation and driving SGs formation, closely linked to eukaryotic translation initiation. In mammalian cells, ISR is regulated by four eIF2α kinases: protein kinase R (PKR), PKR-like endoplasmic reticulum kinase (PERK), general control non-repressible 2 (GCN2), and heme-regulated inhibitor kinase (HRI) [18]. These kinases are activated by various stimuli, phosphorylating eIF2α at serine 51. This blocks the eIF2-GTP-tRNAi^Met^ complex formation, inhibiting mRNA translation and promoting SGs assembly [19].

**Fig. 2:**
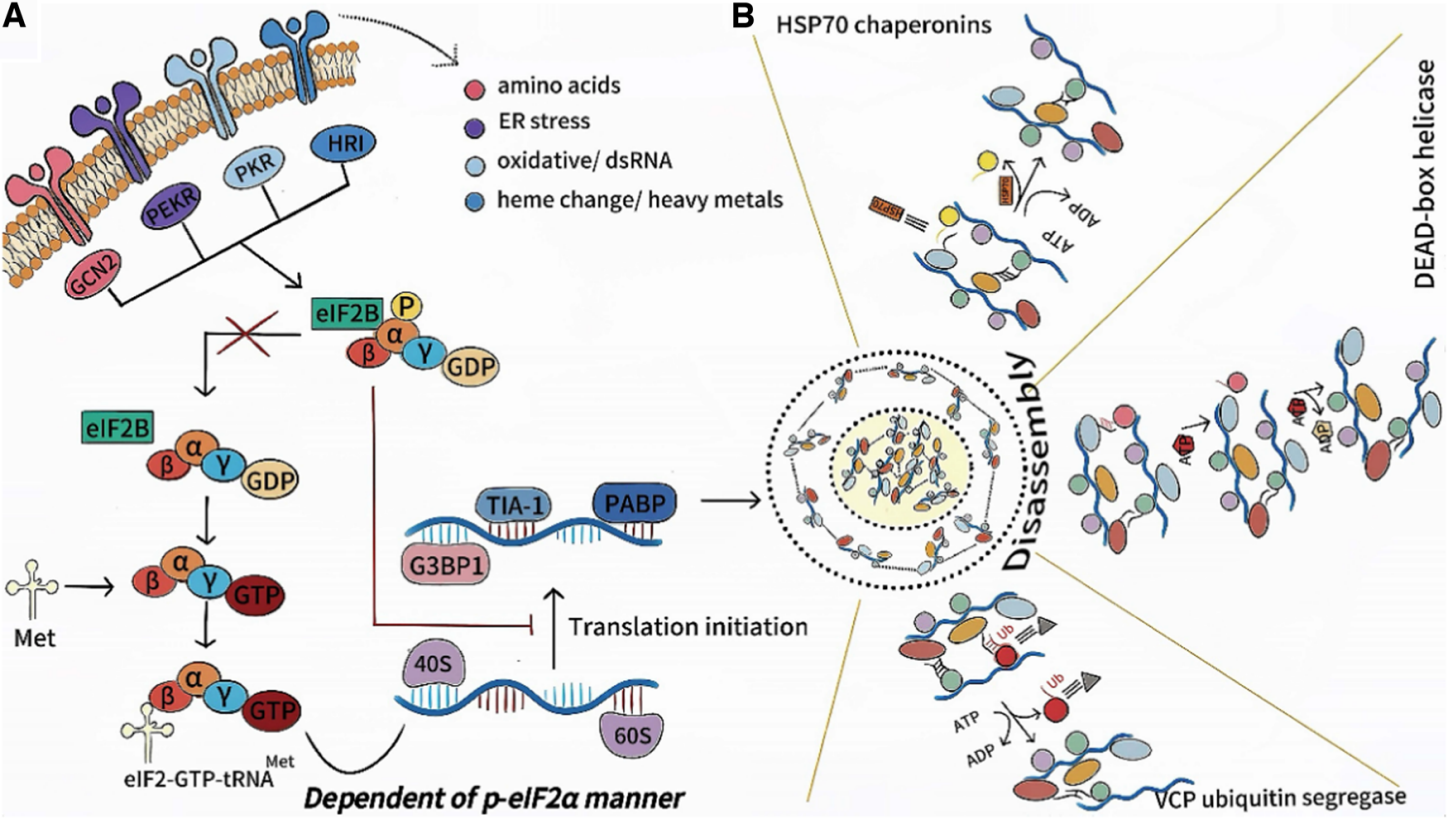
Dynamic assembly and depolymerization of SGs. **A** PKR, PERK, GCN2, and HRI kinases, activated by various stimuli, phosphorylate eIF2α at Serine 51. This blocks eIF2-GTP-tRNAi^Met^ ternary complex formation and triggers SGs assembly. **B** Heat shock proteins, helicases, and VCP all influence SGs assembly by harnessing the energy of ATP hydrolysis to reshape specific interactions. (Created with huashijie.art).

SGs disassembly mechanisms are still underexplored. SGs are highly dynamic and rapidly disassemble within minutes after stress relief, releasing the mRNP complex and restoring protein synthesis [20]. This process is mainly mediated by proteasome degradation and autophagy, involving multiple cellular components, as shown in Fig. 2. Heat shock proteins (HSPs) recognize and bind to misfolded or aggregated proteins, promoting their correct folding or degradation to drive SGs disassembly [21]. EIF2α phosphorylation directly regulates SGs assembly and disassembly, with its dephosphorylation being a key step in dismantling [22]. Additionally, microtubule network reorganization provides structural support for SGs and participates in their transport and degradation [23].

Other cellular components also aid SGs disassembly. Valine-containing proteins (VCPs) break down protein aggregates to facilitate SGs disassembly [21]. Autophagy proteins (e.g., CALCOCO 2, SQSTM1) clear SGs components via autophagy [24], while molecular chaperones, like HSP70, assist in protein folding and transport [25]. Additionally, ATPase provides energy for interactions between chaperones and RNA helicases, promoting SGs component dissociation and transport. RNA helicases unravel RNA structures to release mRNA and restore translation [26]. Dysfunction of these components delays SGs disassembly.

Future research should clarify these cellular components cooperative mechanisms to fully understand SGs dynamics and functions in stress responses, offering potential new insights for disease treatment.

### Functional and pathological significance of SGs

Initially, SGs were thought to temporarily store untranslated mRNAs to prevent degradation and mistranslation under stress, and release mRNA and proteins after stress relief to restore translation and normal function [27]. However, further studies reveal that SGs have more complex and diverse functions.

SGs play a dynamic role in translation regulation. Studies show that mRNAs shuttle between SGs and the cytoplasm, allowing SGs to quickly respond to environmental changes through selective mRNA translation [28]. This enables cells to flexibly adjust protein synthesis under stress, maintaining survival. Additionally, SGs aggregate key molecules to maintain cellular homeostasis and resist damage [29]. They also sequester signaling molecules like TOR, RACK1, and TRAF2, helping cells regain function post-stress [30]. For example, Hu et al. found that SGs protect tumor cells during chemotherapy, providing new ideas for anticancer drug development [29].

In conclusion, SGs are crucial for mRNA storage, translational regulation, stress adaptation and signal transduction. Future studies will further reveal their complex functions and mechanisms, offering new insights into cellular stress responses and related diseases.

### The role and significance of SGs in infectious diseases

#### SGs formation in infectious diseases

In infectious diseases, pathogens intricately regulate SGs formation and breakdown. Host cells activate the ISR through eIF2α phosphorylation, driving SGs formation [4]. Disruptions in protein synthesis, both host and viral, also trigger SGs assembly by causing mRNP complexes to aggregate [31]. Various viruses modulate SGs dynamics through specific mechanisms. For example, respiratory syncytial virus (RSV), Sendai virus, enterovirus type 71 (EV71), polio virus (PV), and encephalomyocarditis virus (EMCV) induce early SGs formation by upregulating G3BP1 and phosphorylating eIF2α [32, 33]. However, viruses such as PV and EMCV later degrade G3BP1 to inhibit SGs assembly, enhancing viral replication [34]. Additionally, viruses like Middle East Respiratory Syndrome Coronavirus, Severe acute respiratory syndrome Coronavirus type 2 (SARS-CoV-2) and Japanese Encephalitis virus (JEV) basically inhibit SGs formation to evade host immune surveillance [4, 35].

Research on non-viral pathogens is relatively limited, but available evidence suggests that bacteria and protozoa can modulate SGs assembly and disassembly through specific mechanisms. Bacteria influence eIF2α phosphorylation to promote or inhibit SGs formation [36]. For example, *Shigella* infection disrupts SGs formation by blocking eIF3 and eIF4B recruitment and inhibiting G3BP1 and eIF4G aggregation, affecting eIF2α phosphorylation or eIF4A helicase activity. In *Listeria*-infected cells, elevated eIF2α phosphorylation is linked to SGs formation [4]. *Mycobacterium tuberculosis* (Mtb) significantly induces SGs formation. Studies have shown that SGs aggregate around infection sites in Mtb Erdman-infected mouse macrophages [37]. Additionally, wild-type Mtb-infected mouse show SGs, absent in ESX-1 mutant-infected mice. Similarly, wild-type Mtb induces SGs near membrane damage in human macrophages. These findings suggest that SGs formation is a critical host response to Mtb infection [38]. Protozoa interact with host cells by influencing SGs formation. *Plasmodium* promotes SGs formation by disrupting host translation, potentially aiding pathogen resistance [39]. In *Trypanosoma brucei* (T.brucei), ATP levels correlate with SGs composition and dynamics, and SGs formation helps it adapt to environmental changes like glucose depletion by altering mRNA and protein profiles [40].

#### Pathogen utilization and regulation of SGs

SGs are key host defenses against pathogens. However, many pathogens manipulate SGs formation and function to evade immunity and promote infection [41]. These strategies mainly include: (1) inhibiting PKR activation by hiding or degrading double-stranded RNA (dsRNA); (2) cleaving key SGs components; (3) redirecting SGs-related factors to viral replication complexes.

**Fig. 3:**
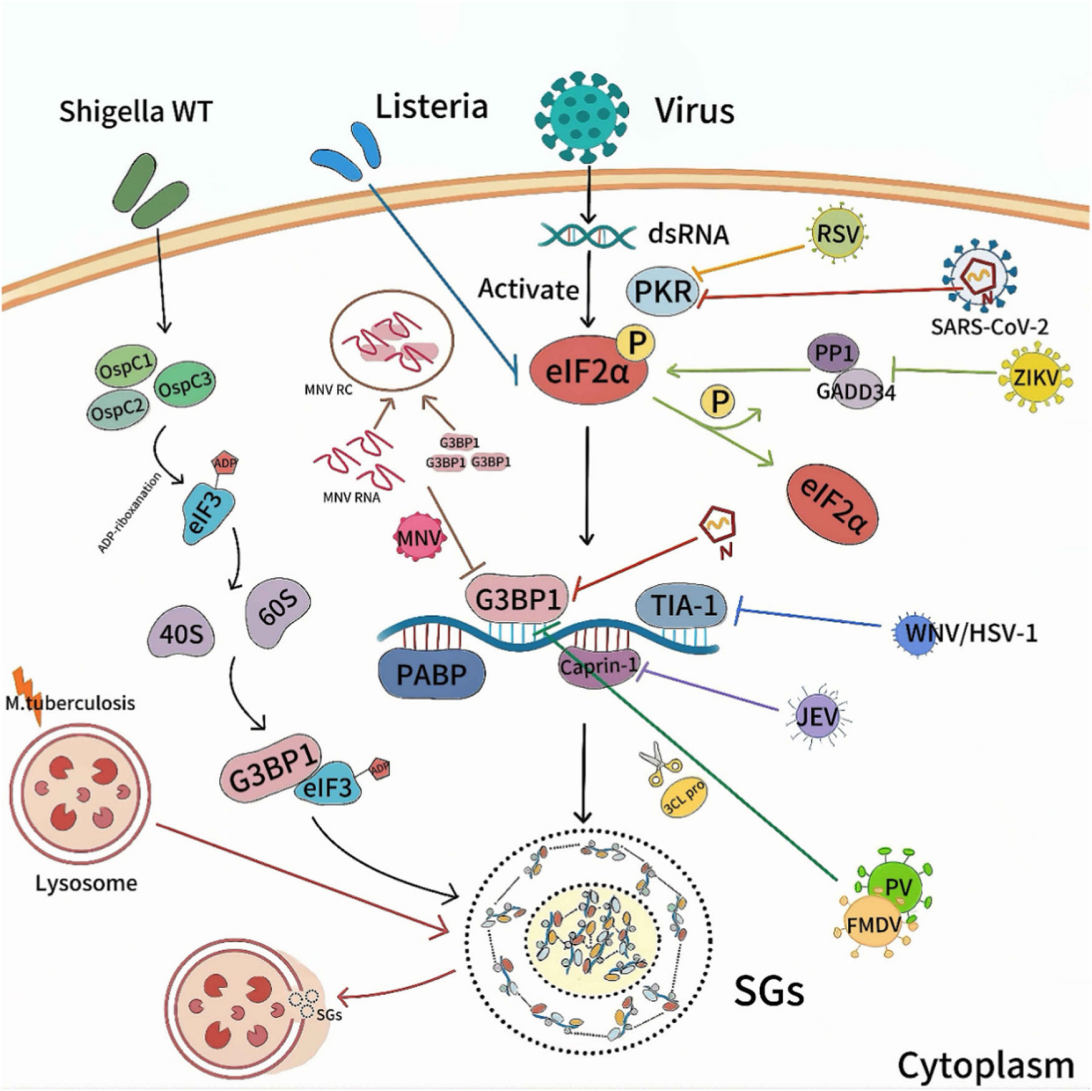
Interactions between SGs and pathogens. Vrial dsRNA actives PKR to phosphorylate eIF2α, promoting SGs formation; however, many virus(e.g., SARS-CoV-2, ZIKV, FMDV, PV, MNV, RSV, WNV, HSV-1, JEV) use diverse strategies to manipulate SGs, evading host immunity and enhancing replication and transmission, while some bacteria (e.g., Shigella, Listeria, Mtb) can also regulate SGs assembly through various strategies. (Created with huashijie.art).

Viruses counteract SGs formation using various tactics, as shown in Fig. 3. Influenza virus induces ER stress (ERS), primarily activating IRE1, a key regulator that supports viral protein synthesis and replication while affecting SGs dynamics [42]. It also can recruit P58IPK to inhibit PKR activity, reducing PKR and eIF2α phosphorylation and increasing viral protein levels [43]. Additionally, its NS1 protein blocks PKR activation by inhibiting dsRNA binding [44], and P58IPK further suppresses PKR to aid viral evasion of host defenses [45]. The VP35 protein of Ebola virus (EBOV) can interfere with the aggregation of SGs-related proteins at a high expression level and disrupt the formation of SGs [46]. Zika virus (ZIKV) upregulates GADD34 to promote eIF2α dephosphorylation [41, 47]. SARS-CoV-2 nucleocapsid protein (NP) inhibits PKR activation and binds to G3BP1’s NTF2L domain to promote viral replication [48, 49]. Viral proteases from viruses like FMDV and PV cut G3BP1 late in infection [32, 50]. Mouse norovirus (MNV) recruits G3BP1 to its replication site, where G3BP1 binds viral RNA via its RNA recognition motif, promoting viral replication and blocking SGs formation [51].

Whether bacteria and parasites manipulate SGs formation and dynamics is still under investigation. Recent studies suggest that *Shigella flexneri* uses its OspC effector to promote SGs formation via eIF3-G3BP interactions, supporting bacterial survival and virulence [52]. *Coxiella burnetii* (C. burnetii) uses type IV secretion system (T4SS) to deliver effector proteins that inhibit SGs formation and evade host stress response [53]. Meanwhile, it activates the host’s unfolded protein response (UPR) and eIF2α pathway, potentially promoting SGs formation [54]. However, the specific mechanisms of C. burnetii on SGs formation and assembly remain unclear. Different *Escherichia coli* (*E. coli*) strains regulate SGs differently *E. coli* HS and *enterotoxigenic E. coli* blocks SGs formation by resisting oxidative stress or sodium arsenite treatment, promoting bacterial colonization [55]. However, subtilase cytotoxin from *Shiga-toxigenic E. coli* (STEC) activates the PERK/DAP1 pathway to induce SGs formation [56].

Understanding how pathogens manipulate host cell stress responses requires analyzing their impact on SGs formation. Table 1 shows how pathogens affect SGs formation, impacting their life cycles and host defenses.

**Table 1:** Mechanisms of induction or inhibition of SGs formation by different pathogens.

Pathogen	Category	Induce	Inhibit	Mechanism	References
Virus	ZIKV		√	Up-regulated GADD34 expression or NS2B mediates the interaction between Protein phosphatase 1α (PP1α) and eIF2α to promote the dephosphorylation of eIF2α	[41]
	SARS-CoV-2		√	Nucleocapsid protein (NP) inhibits the phosphorylation and activation of PKR and binds to the NTF2L domain of G3BP1 to further promote viral replication	[48, 49]
	Influenza virus	√		Induce ERS-mediated phosphorylation of eIF2α	[151]
			√	Recruit P58IPK within host cells to downregulate PKR activity	[43]
				NS1 protein inhibits PKR activity	[44]
	EBOV		√	VP35 protein interfere with the aggregation of SGs-related proteins	[46]
	EV71	√		2 A protease cleavage of eIF4GI induces the formation of atypical stress particles (aSGs)	[32]
	FMDV		√	Leader protease or 3 C protease cuts G3BP1	[50, 152]
	PV	√		Upregulated expression of G3BP1 and phosphorylation of eIF2α	[153]
			√	3 C protease cuts G3BP1	[32]
	WNV		√	TIA-1 is intercepted at the 3’end of the viral genome	[154]
	JEV		√	The core protein interacts with Caprin1	[155]
	RSV	√		Activate the PKR pathway	[156]
	SeV	√		Activate the PKR pathway	[33]
	MRV	√		Early infection promotes eIF2α phosphorylation	[157]
			√	Non-PKR dependent, inhibit SGs generation	[158]
	MNV		√	G3BP1 is recruited to the replication site of viral RNA and binds to it	[51]
	HSV-1	√		TIA-1 was intercepted by RNA stem loop structure	[159]
			√	Loss of Vhs can induce the production of SGs	[160]
Bacteria	Shigella flexneri	√		The interaction between eIF3 and G3BP was induced by Osp C effectors	[52]
	Listeria	√		Promote phosphorylation of eIF2α	[4]
	C. burnetii	√		May promote phosphorylation of eIF2α	[54]
			√	Use type T4SS to deliver effector proteins that may influence SGs formation	[53]
	Escherichia coli	√		The induced production of new RNA cirglis3 (cGLIS3) regulates the interaction and ubiquitination ofhnRNPA1 and G3BP1	[63]
				Activate the PERK/DAP1 signaling pathway	[56]
			√	Resistance to oxidative stress or sodium arsenite treatment	[55]
Protozoa	Plasmodium	√		By interfering with the host’s translation mechanism	[39]
	T. brucei	√		ATP levels affect the formation and composition of SGs	[40]

#### Roles of SGs in infectious diseases

The ISR, especially SGs formation, is vital for antiviral immunity. After viral infection, the host immune system recognizes viral nucleic acids through pattern recognition receptors (PRRs), activating downstream pathways and antiviral responses. During RNA virus replication, dsRNAs are recognized by RLRs, which transmit signals via mitochondrial antiviral signaling proteins (MAVS) [57]. This process is linked to SGs dynamics. Viral nucleic acids activate PKR-mediated eIF2α phosphorylation, promoting SGs formation, inhibiting translation, and blocking viral protein synthesis [58]. Study shows that silencing G3BP1 or PKR reduces SGs formation in influenza A virus (IAV)-infected cells, highlighting SGs’ role in maintaining RIG-I signaling and antiviral responses [59]. TRIM25 co-condenses with G3BP1 in SGs to enhance its ubiquitination activity, which is vital for activating the RIG-I pathway and restricting RNA virus infection [60]. Additionally, zinc finger CCCH-type antiviral protein ZC3HAV1 inhibits viral replication by degrading viral mRNA and collaborates with MOV10, ZCCHC3, and other factors to regulate SGs formation [61].

**Fig. 4:**
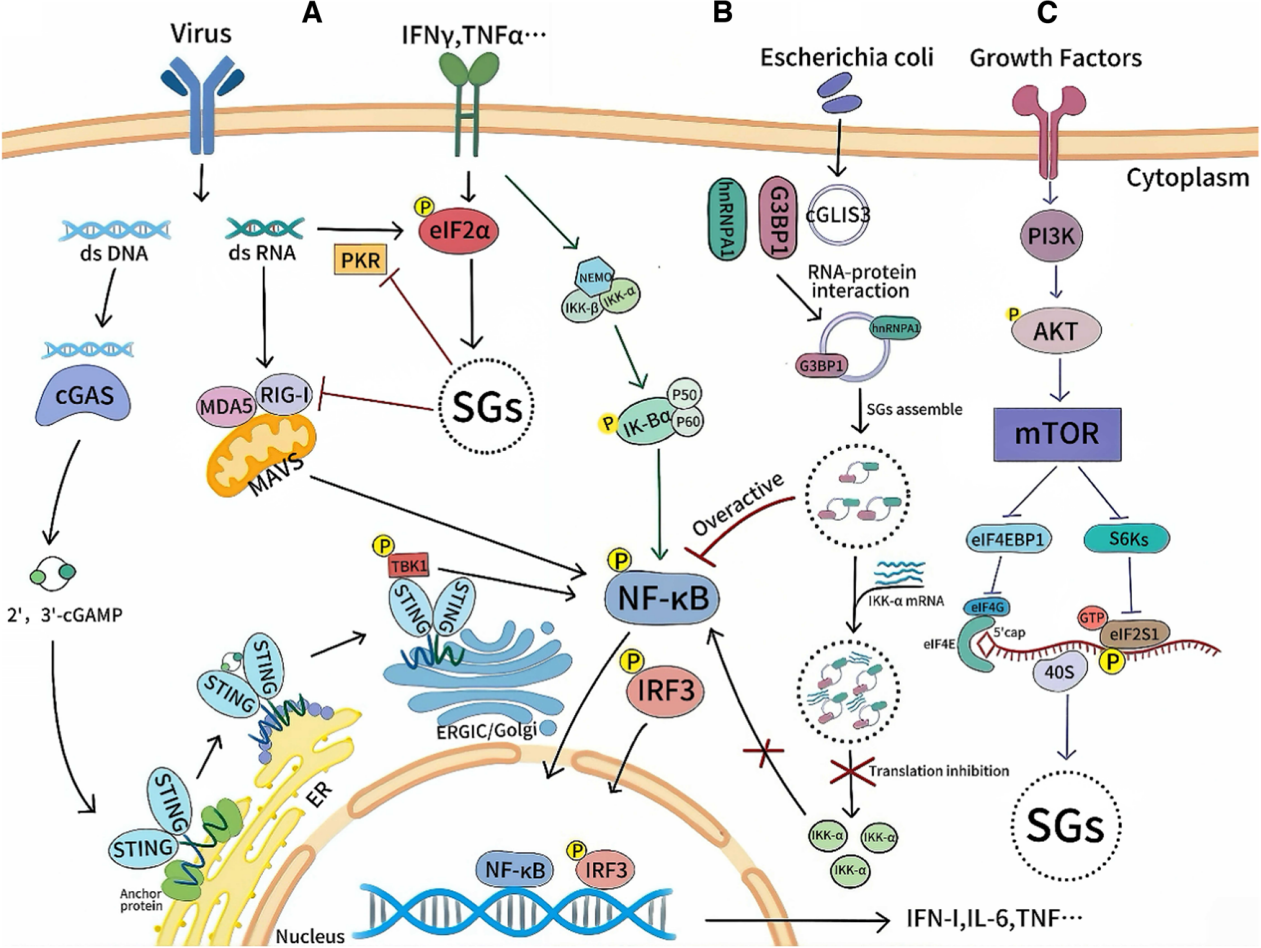
SGs are involved in regulating signal transduction as a signal center. **A** Antiviral immune pathway. (a) cGAS detects viral dsDNA, produces cGAMP to activate STING, which triggers TBK1 to phosphorylate IRF3, promoting its nuclear translocation and IFN expression. (b) RNA virus dsRNA is recognized by RIG-I and MDA5, with signaling transmitted via MAVS. (c) Viral nucleic acids trigger PKR-mediated eIF2α phosphorylation, driving SGs formation. **B** SGs and NF-κB pathway: E. coli induces cGLIS3, which promotes SGs assembly via hnRNPA1-G3BP1 interaction and ubiquitination, reducing IKKα and aberrantly activating the NF-κB pathway. **C** SGs-mTORC pathway: under stress, the mTOR-S6K axis enhances eIF2α phosphorylation, or mTOR phosphorylates eIF4EBP1 to trigger SGs assembly. (Created with huashijie.art).

SGs also regulate cell growth, differentiation, and death. Loss of SGs can overactivate the RLR pathway, induce overexpression of pro-apoptotic genes (e.g., TNF-α, FAS), and cause cell death. Thus, SGs act as a “shock absorber” to maintain cell homeostasis by regulating immune responses to viral dsRNA and preventing cytotoxic overreaction [62]. Under conditions such as Mtb infection, SARS-CoV-2 ORF3a expression, and tau protein aggregation, SGs nucleate near damaged lysosomes, acting as “safety plugs”. They stabilize ruptured lysosomal membranes, promote membrane repair, and prevent cell death [38]. SGs also regulates inflammation by controlling the translation of NF-κB pathway-associated proteins. For example, E. coli-induced cGLIS3 promotes SGs assembly by modulating hnRNPA1-G3BP1 interactions and ubiquitination, blocks IKKα mRNA, activates NF-κB, and drives tumor resistance and intrahepatic cholangiocarcinoma metastasis [63]. Figure 4 shows pathways to SGs formation from viral infections, bacteria, and growth factors, highlighting key molecules and interactions for cellular response regulation.

After analyzing the formation mechanism, components and role of SGs in infectious diseases, we inevitably focus on its close connection with the fate of cells. SGs protects host cells under moderate stress, but when the stress exceeds the regulatory capacity of the cells, cell death becomes an inevitable outcome. In the physiological and pathological processes of cells, SGs does not exist and function in isolation. It is intertwined and interacts with the mechanism of cell death, jointly shaping the course and outcome of infectious diseases. Next, this article will delve into the role of the cell death mechanism in infectious diseases and its intricate interrelationship with SGs.

## Role of cell death mechanisms in infectious diseases

### Interaction between cell death and immune response

Damage-Associated Molecular Patterns (DAMPs) are host cell components normally found in intracellular or extracellular matrix, including HMGB1, ATP, HSPs, CRT, uric acid crystals, DNA, and RNA. They are released during cell damage, stress, or death, and recognized by PRRs as “danger signals” to activate immune and inflammatory responses [64]. Macrophages and dendritic cells (DCs) recognize DAMPs, secrete inflammatory factors and chemokines, recruit immune cells to the injured site, and clear pathogens and debris. Mature DCs also activate T cells to initiate adaptive immunity [65].

The immune system can also actively regulate cell death to maintain tissue homeostasis and host defense. For example, Cytotoxic T cells and NK cells induce apoptosis by releasing perforin and granase [66]. Macrophages promote cell death by releasing TNF-α and NO [67]. IFN-γ upregulates pro-apoptotic proteins like Fas and Bax to induce apoptosis [68]. This bidirectional mechanism in infectious diseases helps clear pathogens but may also cause excessive immune responses and tissue damage.

### Mechanism and characteristics of pyroptosis

Pyroptosis is a highly inflammatory programmed cell death driven by inflammasome activation, Gasdermin D (GSDMD) cleavage, and cell membrane perforation. Inflammasome activation initiates this process, characterized by cell membrane rupture, inflammatory cytokine release, and intercellular transmission, which are crucial for inflammation and pathogen clearance [69]. The inflammasome is a multiprotein complex composed of PRRs (e.g., NLRP3, AIM2, NLRC4), ASC, and pro-caspase-1, recognizing PAMPs or DAMPs to trigger innate immune responses [70]. After the inflammasome is activated, caspases (e.g., Caspase-1/4/5/11) cleave pro-IL-1β and pro-IL-18 into mature forms, while cutting GSDMD to generate an active N-terminal fragment (GSDMD-N), which forms pores in the cell membrane, causing cell rupture and release of contents [69].

DAMPs and inflammatory factors released during cell membrane rupture can recruit immune cells, enhance immune response [71], and induce pyroptosis in neighboring cells via extracellular vesicle-mediated GSDMD pore transfer, creating a “bystander effect” [72]. Moderate pyroptosis prevents pathogen spread by lysing infected cells, as seen in infections like *salmonella*, *Listeria*, and influenza virus [69]. Additionally, PITs formed during pyroptosis can trap microbes, limiting pathogen spread and promoting neutrophil phagocytosis [73]. However, excessive pyroptosis in conditions like sepsis, cancer, and pulmonary tuberculosis can cause tissue damage, increased inflammation, and neuroinflammation, contributing to neuronal death in diseases like Alzheimer’s and Parkinson’s [69]. Thus, precise regulation of pyroptosis is essential to balance pathogen clearance and tissue protection.

### Pathological significance of apoptosis and necroptosis

Apoptosis is an orderly, non-inflammatory process driven by caspase activation, maintaining tissue homeostasis and eliminating abnormal cells. Necroptosis is an inflammatory process relying on the RIPK1/RIPK3/MLKL pathway, involved in immune defense and pathogen clearance [74]. They are two different types of RCD.

When pathogens invade host cells, the host can induce infected cell death via exogenous or endogenous apoptotic pathways to clear the infection and limit pathogen spread. For instance, Shiga toxins from STEC and SARS-CoV proteins 3a/7a interact with Bcl-2 family proteins to release mitochondrial cytochrome C (Cyt c), activating the endogenous caspase pathway [75, 76]. IAV infection upregulates Caspase-8 mRNA expression, activating the exogenous apoptotic pathway [77]. Additional, macrophage phagocytosis of apoptotic cells can sometimes induce immune tolerance to avoid excessive immune responses [78]. Conversely, pathogens can also inhibit apoptosis to promote their own replication. After infecting macrophages, C. burnetii activates the eIF2α pathway, increasing ATF4 levels and inducing CHOP expression. However, it inhibits CHOP nuclear translocation via T4SS to prevent apoptosis [54]. It also blocks caspase-3 cleavage and activates the MAPK survival pathway, thereby promoting its own replication [79].

Necroptosis is highly immunogenic, driven by the RIPK1/RIPK3/MLKL axis. Upon receiving a death signal, RIPK1 activates RIPK3, which phosphorylates MLKL, causing it to oligomerize and insert into the cell membrane, increasing permeability and triggering necroptosis [80]. Some viruses, like influenza viruses and other myxoviruses activate ZBP1 via Z-RNAs to induce RIPK3-dependent necroptosis, limiting viral replication [81]. However, many viruses have evolved strategies to evade necroptosis. For example, HSV-1/2 evade necroptosis by using ICP6 and ICP10 proteins to block RIPK1-RIPK3 interaction [82]. In bacterial infections, necrotic apoptosis can be both protective and pathogenic. *Enteric pathogenic E. coli* secretes NleB1 to inhibit necroptosis, and strains lacking NleB1 cannot colonize intestinal epithelial cells [83]. Conversely, *Salmonella* inducing necroptosis in intestinal epithelial cells, disrupting the intestinal barrier and facilitating its invasion [84].

**Fig. 5:**
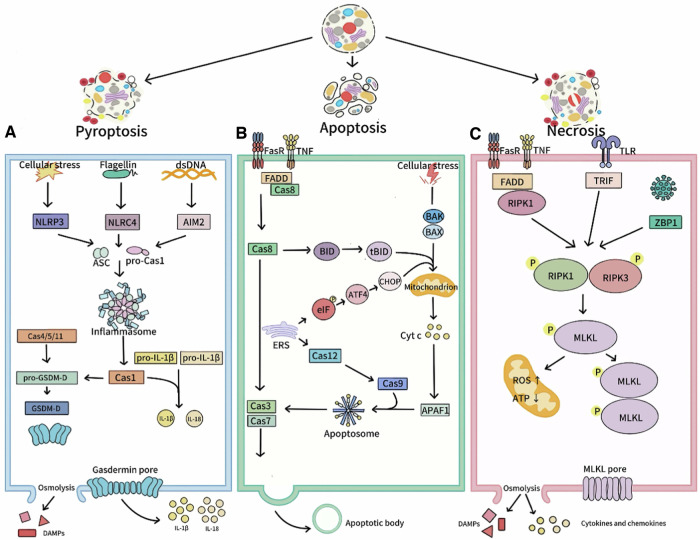
Signaling pathways of pyroptosis, apoptosis, and necroptosis. **A** Pyroptosis: inflammasome activation enables caspase to cleave pro-IL-1β/18 and GSDMD, forming pores and causing cell rupture and content release. **B** Apoptosis: (a) Endogenous: stress signals boost pro-apoptotic proteins, increase mitochondrial permeability, release Cyt c, form apoptosomes with Apaf-1, activate caspases, inducing apoptosis. (b) Exogenous: death receptor-ligand binding recruits FADD, activates caspase-8, triggering downstream caspases or cleaving Bid to induce apoptosis. (c) ERS activates IRE1, PERK, and ATF6 pathways, leading to caspase-12/9 activation and endogenous apoptosis. **C** Necroptosis: death receptor-ligand binding or ZBP1 sensing viral nucleic acids activates the RIPK1/RIPK3/MLKL axis, leading to mitochondrial changes, MLKL oligomerization, and cell rupture. (Created with huashijie.art).

Cell death is crucial for pathogen removal and tissue protection. Figure 5 summarizes the signaling pathways of pyroptosis, apoptosis, and necroptosis, helping clarify their roles in infectious diseases and supporting disease prevention and treatment.

### Recent advances in cell death research

In addition to pyroptosis, apoptosis, and necroptosis, immunogenic cell death (ICD), PANoptosis, ferroptosis, and cuproptosis have gained attention for their unique functions and clinical potential.

ICD triggers immune responses through pathways like apoptosis to clear damaged or infected cells. It requires antigenicity, adjuvants, and a suitable microenvironment [85]. ICD can both activate immune cells and cause immune depletion, as seen in HIV infection [86]. Pathogens like influenza virus (activating the ZBP1/DAI-dependent NLRP3 inflammasome) and *Staphylococcus* aureus toxin (inducing necrosis in immune cells) can induce ICD [87]. ICD is crucial in tumor immunotherapy, with novel inducers like circular RNA cEMSY and oncolytic viruses enhancing antitumor immunity by releasing immune-stimulating molecules during cell lysis [88, 89].

PANoptosis is an inflammatory programmed cell death pathway that integrates pyroptosis, apoptosis, and necroptosis, mediated by the PANoptosome complex. During infection, PAMPs activate PANoptosome to trigger cell death [90]. For example, influenza virus induces ZBP1-PANoptosome formation and promots NLRP3 activation and cell death [90, 91]. SARS-CoV-2 activates JAK/STAT1/IRF1 pathway, inducing Caspase-8-FADD-related PANoptosis and NO production [92]. In addition, elevated ZBP1 in COVID-19 may worsen outcomes via PANoptosis and cytokine storms, suggesting potential therapeutic value in inhibiting PANoptosis [93]. In bacterial infections, *Yersinia pestis* induces RIPK1-PANoptosome complex, regulating cell death [94]. When *Enterococcus* infects macrophages, panoptosis-related genes and proteins trigger multiple cell death modes [95]. PANoptosis is significant in infectious diseases, and its regulatory mechanisms and therapeutic potential warrant further investigation.

Ferroptosis induces lipid peroxidation, damaging cell membranes and causing cell death, restricting pathogen replication and directly damaging pathogens via lipid peroxides and reactive oxygen species (ROS) [96]. In viral infections, Hepatitis B virus induces ferroptosis in hepatocytes by inhibiting SLC7A11 expression, causing liver failure, while SLC7A11 overexpression or ferroptosis inhibitors can rescue these cells [97]. In COVID-19 patients, altered ferroptosis-related gene expression suggests ferroptosis may contribute to tissue damage [98]. Mtb induces ferroptosis by reducing GPX4 expression, aiding its survival and worsening infection [99]. *Pseudomonas aeruginosa*’s lipoxygenase can directly induce ferroptosis [100]. Ferroptosis also modulates the immune microenvironment. Selenium enhances GPX4 in T helper cells (Tfh), protecting Tfh cells from ferroptosis and boosting humoral immunity [101].

Cuproptosis is cell death induced by copper ions binding to mitochondrial lipoacylated proteins, causing protein aggregation, iron-sulfur cluster dysfunction, and cytotoxic stress [102]. It disrupts bacterial respiratory chains, showing strong antibacterial effects on various microorganisms. A copper-manganese nanoparticle can kill bacteria, destroy biofilms, reduce virulence, and improve mouse survival rates [103]. Cuproptosis also activates the immune response by releasing DAMPs and tumor antigens, inhibits tumor progression by regulating tumor cell metabolism and mitochondrial function, and remodels the tumor microenvironment. It enhances anti-tumor effects by affecting tumor angiogenesis and immune cell infiltration [104]. However, elevated copper levels can promote PD-L1 expression, aiding tumor immune evasion [105].

Cell death mechanisms also play key roles in fungal infections. *Candida albicans* and *Aspergillus* induce macrophage death and cytokine release through ZBP1-mediated PANoptosis to control infections [106]. Additionally, *Candida albicans*’ Als3 protein, when internalized by macrophages, activates the inflammasome, induces pyroptosis, and drives immune evasion and local inflammation [107]. Ferroptosis is also involved, with PPZ1 loss in *Candida albicans* increasing sensitivity to ferroptosis inducers [108]. New study suggests using Cu^2+^-based nanoflowers to combat fungal infections through copper toxicity and cell wall digestion [109].

## The interaction between SGs and cell death

In infectious diseases, SGs interact with cell death mechanisms, influencing host stress and immunity, and shaping infection outcomes.

### The dual role of SGs in cell death

SGs inhibit non-essential mRNA translation to save energy and support repair under mild stress, but can trigger cell death via abnormal assembly or accumulation under intense stress. This dual role makes SGs a key regulator of cell fate, offering insights into infectious disease pathology.

SGs inhibit cell death by sequestering key components of pro-apoptotic pathways. For example, RBM3 promotes SGs formation to prevent neuronal apoptosis [110], while TRAF2 and RACK1 recruitment into SGs blocks TNF signaling and caspase-3 activation, respectively, reducing inflammation and apoptosis [111, 112]. In Coxsackie virus B3 infection, SGs reduce ROS production to inhibit apoptosis [113]. Additionally, SGs sequester DDX3X, suppressing NLRP3 inflammasome activation and pyroptosis [114].

Under chronic stress, persistent SGs form due to improper depolymerization or clearance. They inhibit translation, disrupt mRNA regulation, and impair cellular energy metabolism and signaling [115]. Persistent SGs may aggravate ERS, continuously activating the UPR and ultimately inducing cell death [116]. They can also inhibit autophagy, hindering the clearance of damaged organelles and protein aggregates, worsening cellular stress and causing cell death [117]. Genetic mutations, abnormal protein deposits, or other pathological factors can induce the formation of more stable and pathogenic SGs. For example, the CMT2 mutant protein enters SGs and binds to G3BP, disrupting their function and increasing motor neuron vulnerability [118]. In amyotrophic lateral sclerosis and Alzheimer’s disease, SGs accumulation correlates with Tau or TDP-43 aggregation, impairing cell function and survival [19]. These findings highlight SGs’ dual role in cellular stress responses and their potential as therapeutic targets in infectious and degenerative diseases.

### Impact of cell death on SGs

Cell death regulates SGs dynamics bidirectionally. Viral nucleic acid from dead cells activates PKR-mediated eIF2α phosphorylation to promote SGs formation. Similarly, inflammatory factors like IFN-γ and TNF-α induce eIF2α phosphorylation to drive SGs assembly [119]. In atherosclerosis, SGs markers are upregulated in endothelial cells, macrophages, and smooth muscle cells, correlating with disease progression. Oxidized low-density lipoprotein (oxLDL) and mitochondrial stress induce PABP^+^ and G3BP1^+^ SGs in these cells. Notably, IL-19 inhibits SGs formation, reduces PABP and eIF2α phosphorylation, and may help control vascular inflammation [120].

However, apoptotic signals or lysosomal enzyme release degrade SGs components, inhibiting formation or promoting decomposition. HSPs help correct protein folding and dissolve SGs, while ATPase powers chaperones and RNA helicases to facilitate SGs disintegration. This process affects cell fate in two ways: First, mRNAs released from SGs may be degraded or re-regulate cell death-related gene expression [121]. Second, RNA and proteins in SGs may be released outside the cell after cell membrane rupture, acting as DAMPs to activate the immune system and trigger inflammation. In addition, Cell death can terminate SGs’ protective function. SGs need cellular energy and resources to form and maintain, and autophagy, lysosome, and VCP dysfunction during cell death can impact their morphology and function [122]. In stressed T. brucei, extracellular carbon and ATP levels affect SGs’ composition and dynamics, showing that energy levels are crucial for SGs, and energy disruption can cause dysfunction.

### Synergistic mechanism between SGs and cell death and its significance in infectious diseases

The SGs-cell death synergy is a complex, dynamic process, centrally regulating stress response and homeostasis via shared signaling pathways.

In early ERS, IRE1α splices XBP1 mRNA to generate XBP1s protein, regulates ER gene expression, alleviates ERS, and maintains cell survival [123]. However, persistent or excessive ERS causes IRE1α to bind to TRAF2, activating the JNK pathway and inducing apoptosis [124]. IRE1α aggregation dynamically combines with SGs to form IRE1α-SGs co-aggregate, crucial for IRE1α signal activation and XBP1 mRNA splicing. SGs integrity also affects IRE1α aggregation formation and function [116]. ASK1 is a core regulator of the cellular stress response network. It regulates cell proliferation, differentiation, and apoptosis via JNK and p38 MAPK phosphorylation [125]. JNK and p38 activation also promotes SGs formation, influencing cell survival fate [126, 127]. Under hypertonic stress, cells increase intracellular pH through a sodium/hydrogen exchanger, activating RIPK3/MLKL-mediated cell death [128]. In ISR, eIF2α phosphorylation inhibits translation to relieve stress. Both conditions induce SGs, which protect cells early on, but their accumulation under sustained stress causes metabolic disorder and cell death [129, 130]. This transition mechanism reveals a fine regulatory network of cellular stress responses, offering potential therapeutic targets for infectious diseases.

In infectious diseases, SGs inhibit pathogen translation to block infection, while cell death clears infected cells and activates innate immunity. These processes shape disease outcomes and host defense. However, many pathogens have evolved multiple strategies to disrupt this balance. Different viruses employ various strategies to evade host defenses and enhance their replication. Human parainfluenza virus (HPIV3) hides its RNA in inclusion bodies to inhibit SGs formation [131], while inducing mitochondrial autophagy to suppress interferon responses and boost viral replication [132]. PV inhibits SGs formation by degrading G3BP1 through its 3C protease, while using VP3 protein to induce apoptosis and necrosis to promote viral release [133]. In bacterial infection, pathogens secrete effector proteins to disrupt SGs formation or function, indirectly regulating apoptosis. *Legionella pneumophila* secretes LegK3 to inhibit caspase activity [134], preventing apoptosis and using the host stress response to boost its own replication [135]. Mtb damages lysosomal membranes in macrophages, triggering SGs formation to limit infection. Its Mce3C protein targets lysosomal proteases, inhibits apoptosis, and promotes necroptosis, enhancing intracellular survival and host inflammation [136].

Current research on whether parasites disrupt the balance between SGs and cell death is limited. Oxidative stress is key in parasitic infections, with host cells producing ROS to combat infection while parasites develop antioxidant defenses. ROS can induce SGs formation to protect cellular components, but excess ROS can cause cell damage and death by disrupting redox balance [137]. For instance, ROS oxidizes TIA1, inhibiting SGs assembly and promoting apoptosis [138]. This suggests parasites may regulate host stress and cell death for survival, but their role in disrupting the SGs–cell death balance is still unclear and requires further investigation.

**Fig. 6:**
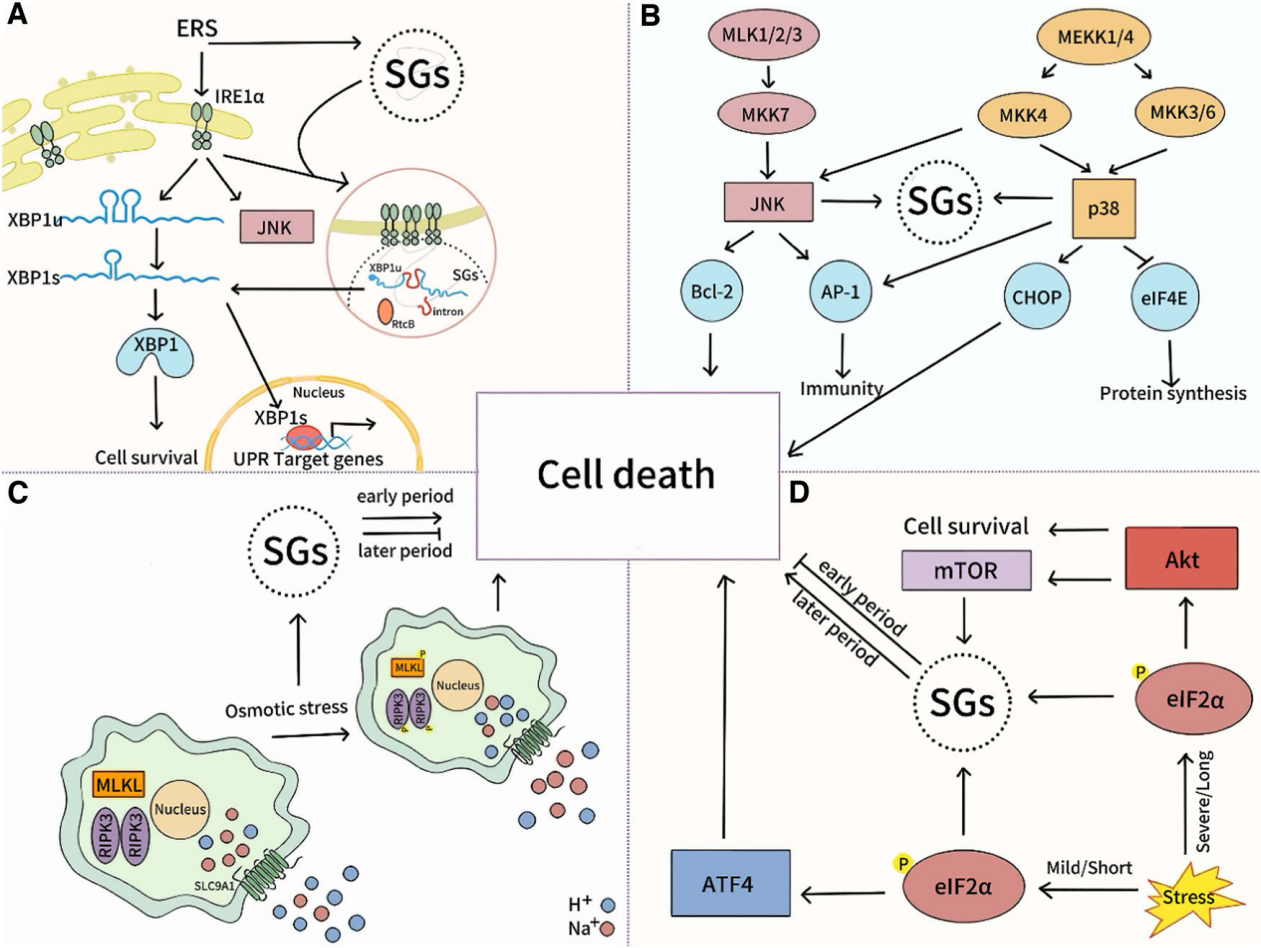
Shared pathways between cell death and SGs. **A** In early ERS, IRE1α splices XBP1 mRNA to produce XBP1s protein, promoting cell survival. Excessive IRE1α activation triggers JNK pathway and induces apoptosis. IRE1α also interacts with SGs to form IRE1α-SGs aggregates. **B** ASK1 activation phosphorylates MKK4/7 to activate JNK, targeting Bcl-2 and AP-1. MKK3/6 phosphorylation activates p38, targeting CHOP and AP-1. These pathways regulate apoptosis, inflammation, and SGs formation. **C** Hypertonic stress raises pH, activates RIPK3/MLKL pathway, induces SGs formation to mitigate damage, but severe or prolonged stress can still cause cell death. **D** EIF2α phosphorylation promotes SGs formation, but sustained eIF2α phosphorylation and SGs accumulation can cause metabolic disorders, leading to apoptosis or necrosis.

SGs and cell death are critical in the pathogen life cycle and host immune response, influencing pathogen-host balance and infection progression. Figure 6 illustrates the key signaling pathways between them. Understanding this mechanism provides a basis for developing new infectious disease treatments.

## Therapeutic potential of SGs and cell death

### Therapeutic strategies targeting SGs

SGs assembly and disassembly offer a new approach to control cell fate and treat infections. SGs limit viral replication by isolating viral RNA and proteins, but some viruses disrupt SGs formation to evade host immunity. HPIV3 hides RNA in inclusion bodies to inhibit SGs formation, while SARS-CoV-2 interacts with G3BP1/2 to suppress SGs assembly and promote viral replication. Viruses like PV and EMCV use viral proteases to degrade G3BP1, inhibiting SGs assembly. G3BP1/2 are emerging as potential anti-infective drug targets due to their critical role in SGs formation.

A variety of small molecule drugs have shown antiviral potential by targeting the SGs-related pathways. EIF4A inhibitors irreversibly block eIF4A activity, induce SGs formation, and inhibit viral replication. For example, silvestrol is effective against EBOV [139], and Pateamine A shows strong inhibition in early IAV infection [140]. Additionally, specific inhibitors of the TUDCA and IRE1 pathways significantly inhibit IAV replication [42]. CK2 inhibitors like tetrabromocinnamic acid and silmitasertib may enhance host antiviral ability, but further validation is needed [34]. Small molecule drugs like puromycin boosts SGs by detaching ribosomes from mRNA, while other drugs such as actinomycin and ISRIB prevent SGs formation and may dissolve existing SGs [141, 142]. The inhibitory effect of ISRIB has been confirmed in C. burnetii infection [54]. These mechanisms influence host cell resistance to pathogen infection by targeting eIF2α phosphorylation pathway. Some antibiotics indirectly affect pathogen infection by regulating host cell stress responses. For example, Psammaplysin F reduces SGs by lowering eIF2α phosphorylation, while Gramicidin S decreases SGs formation by creating cell membrane pores and altering intracellular ion balance [36]. However, its specific role in antibacterial infection needs further clarification.

Additionally, SGs are linked to inflammatory and immune regulation. For example, DDX3X, shared by SGs and the NLRP3 inflammasome, regulates SGs formation under stress, protects liver cells from oxidative and inflammatory damage, and has potential as a drug target [114, 143].

### Therapeutics targeting cell death pathways

Therapeutic strategies targeting cell death pathways show broad application prospects in infectious diseases. Some viruses upregulate anti-apoptotic proteins like Bcl-2 to inhibit apoptosis and promote persistent infection. BH3 mimics (e.g., ABT-737) can inhibit Bcl-2 family proteins, restore apoptosis, and clear infected cells [144]. Additionally, some drugs can induce apoptosis in infected cells by targeting intracellular pathways. For example, SMAC mimetics like LCL161 and AEG40730 induce apoptosis in HIV-infected cells by binding to inhibitor of apoptosis proteins, specifically targeting infected macrophages and CD4^+^ T cells without harming uninfected cells [145]. Emricasan, an irreversible pan-caspase inhibitor, has been studied in ZIKA infection and is considered a potential treatment for COVID-19 [146]. In tuberculosis treatment, drugs that activate necroptosis or autophagy enhance host cell clearance of Mtb [147]. Furthermore, anti-PD-1 or anti-PD-L1 monoclonal antibodies can reactivate T cells to boost their ability to kill infected cells, showing potential in treating HIV, tuberculosis, and malaria [148].

### Prospects of combination treatment strategies

Since the formation and regulation of SGs is closely related to multiple cell death pathways, targeting these pathways can enhance anti-infective effects and reduce inflammatory. Targeting eIF2α phosphorylation can regulate both SGs assembly and apoptosis, offering a dual therapeutic benefit [34]. For example, Pifithrin-µ induces SGs formation while causing cell death through a non-apoptotic pathway [149]. SGs also affect cell survival by regulating energy metabolism and oxidative stress, which are closely linked to mitochondrial function. Targeting these pathways can alter pathogen environments enhance pathogen RNA degradation, and promote infected cell clearance through apoptosis or autophagy. Both SGs and cell death can activate host immune. Enhancing interferon signaling and immune cell activation improves infection-fighting ability. Pathogens may inhibit SGs or cell death to evade defenses. Combination therapy targeting multiple pathways reduces pathogen escape. Understanding SGs and cell death mechanisms in infections can guide personalized therapies using new SGs-targeting agents and cell death inducers, enhancing precision.

Combination therapy shows promise in anti-infection but faces many challenges in clinical application. First, precisely delivering drugs to infection sites and targeting SGs and cell death pathways is key. Developing nanocarriers or lipid nanoparticles may improve drug delivery efficiency [150]. Second, combination therapy may cause excessive immune responses or cytotoxicity, requiring optimized dosing and immunomodulators. Finally, SGs and cell death pathways vary by cell type, and CRISPR-based cell-specific gene editing could enhance therapeutic precision.

## Conclusion and perspectives

The role of SGs in infectious diseases and their regulatory mechanisms are increasingly clear. Pathogens manipulate SGs to evade immune surveillance or boost replication, but how they do this, and how SGs interact with other organelles during infection, remain to be explored. Meanwhile, cell death, a key host mechanism for clearing pathogens, has a complex role in infections. It can help the host by clearing infected cells and activating immunity, but pathogens may suppress it to evade immune responses. Excessive cell death can also cause tissue damage. Thus, the specific role of cell death in infections depends on the pathogen, infection site, and host immune status.

SGs and cell death have a complex two-way feedback loop. Cell death triggers SGs formation via inflammatory factors and DAMPs, enhancing stress responses. SGs protect cells by isolating key proteins in cell death pathways, inhibiting cell death and regulating immunity. However, pathogens can exploit this to cause chronic infections or immune escape. Recently, SGs regulation and cell death have become therapeutic targets for infectious diseases. For example, SARS-CoV-2’s NP inhibits SGs formation by binding to G3BP1, promoting viral replication and offering a potential COVID-19 treatment target. Similarly, eIF4A inhibitors used against EBOV and influenza virus highlight the potential of targeting SGs kinetics. Clinically, targeting key SGs components like G3BP1 and DDX3X shows promise. Future research should explore the roles of SGs and cell death in chronic inflammation, cancer, and neurodegenerative diseases to advance precision medicine.

The non-membrane and dynamic nature of SGs complicates their composition analysis. Future research should integrate advanced technologies to elucidate SGs’ dynamic characteristics and roles in cell death and infection. Single-cell sequencing can reveal transcriptome changes and dynamic gene expression patterns in SGs during infection. Super-resolution microscopy can clarify SGs’ intracellular distribution and interactions with other cellular structures. CRISPR technology can be used to precisely knock out or mutate target genes, study the molecular mechanism of SGs, and knock out anti-apoptosis genes to enhance the sensitivity of cells to apoptosis inducers. Integrating multi-omics analyses and interdisciplinary collaborations, such as bioinformatics and proteomics, will clarify the molecular mechanisms of SGs and cell death, speeding up the development of targeted therapies. Future research should enhance cooperation among immunology, molecular biology, and clinical medicine to advance the application of SGs and cell death research in infectious diseases.

Emerging technologies and deepening interdisciplinary collaboration will reveal the complex roles and mechanisms of SGs and cell death in infectious diseases. Targeting SGs homeostasis and cell death mechanisms provides new strategies for antiviral, anti-inflammatory, and anticancer therapies, and may also offer new approaches for treating neurodegenerative and metabolic diseases. Research and application of SGs and cell death have broad prospects, offering innovative solutions for complex infectious diseases and related pathologies.
